# Salvage surgery for recurrent transdiaphragmatic thymoma in a patient not eligible for chemotherapy

**DOI:** 10.1093/jscr/rjae288

**Published:** 2024-05-05

**Authors:** Giulia Fabbri, Nabih Berjaoui, Savvas Lampridis, Rajdeep Bilkhu, Ishaan Chauhan, Ee Phui Kew, Akshay Patel, Andrea Bille

**Affiliations:** Department of Thoracic Surgery, Guy’s and St Thomas’ NHS Foundation Trust, Great Maze Pond, London SE1 9RT, United Kingdom; Department of Thoracic Surgery, Guy’s and St Thomas’ NHS Foundation Trust, Great Maze Pond, London SE1 9RT, United Kingdom; Department of Thoracic Surgery, Guy’s and St Thomas’ NHS Foundation Trust, Great Maze Pond, London SE1 9RT, United Kingdom; Department of Cardiac Surgery, Guy’s and St Thomas’ NHS Foundation Trust, London, United Kingdom; Department of Thoracic Surgery, Guy’s and St Thomas’ NHS Foundation Trust, Great Maze Pond, London SE1 9RT, United Kingdom; Department of Thoracic Surgery, Guy’s and St Thomas’ NHS Foundation Trust, Great Maze Pond, London SE1 9RT, United Kingdom; Department of Thoracic Surgery, Guy’s and St Thomas’ NHS Foundation Trust, Great Maze Pond, London SE1 9RT, United Kingdom; Institute of Immunology and Immunotherapy, University of Birmingham, Vincent Drive, Edgbaston, Birmingham, B15 2TT, England, United Kingdom; Department of Thoracic Surgery, Guy’s and St Thomas’ NHS Foundation Trust, Great Maze Pond, London SE1 9RT, United Kingdom

**Keywords:** thymoma, recurrence, salvage surgery, extra-pleural pneumonectomy, transdiaphragmatic disease

## Abstract

The recurrence rate following thymoma surgery has been reported to be as high as 29%. In cases of localized recurrence, complete resection can result in prolonged patient survival. However, surgery is rarely considered in cases of invasive recurrent thymomas with high disease burden. Here, we present the case of a woman with type B2 thymoma (Masaoka–Koga stage IVa) treated with surgery, chemotherapy, and radiotherapy. The disease recurred 6 years later, with invasion of the left lung and the 12th thoracic vertebra, as well as extension into the retroperitoneum. Due to the development of chemotherapy-associated toxicity, she underwent surgery with complete tumor resection and has remained free of disease at a 12-months follow-up. Radical surgery for recurrent invasive thymoma extending through the diaphragm is a feasible and safe therapeutic option in highly selected patients who are not eligible for systemic treatments.

## Introduction

Thymomas are the most frequent neoplasms of the anterior mediastinum. For patients in whom a complete resection is considered feasible, surgery is indicated as the initial treatment. The recurrence rate after thymectomy varies between 10% and 29%, depending mainly on disease stage and histological type [1]. Surgical resection can prolong survival in patients with localized recurrent disease [2]. However, in case of less resectable disease, chemotherapy and radiation are usually preferred. Here, we report the case of a patient with recurrent thymoma invading the lung, the spine and extending into the abdomen through the diaphragm, who underwent radical surgical resection.

## Case description

A 53-year-old woman with a past medical history positive for myasthenia gravis, autoimmune hepatitis, membranous nephropathy and systemic lupus erythematosus, was diagnosed in 2015 with Masaoka–Koga stage IVa B2 thymoma. She was treated with six cycles of induction chemotherapy with cyclophosphamide, doxorubicin, and cisplatin, followed by surgery, which included thymectomy, multiple wedge resections of lesions in the left lung, partial pleurectomy, as well as pericardial resection and reconstruction with a synthetic mesh. Due to microscopic residual tumor, she received 60 Gy of radiation in 30 fractions, completed in April 2016. Fifteen months later, the tumor recurred in the left hemithorax. Despite five cycles of chemotherapy with carboplatin and paclitaxel, the disease progressed, and the regimen was switched to dendrimer–docetaxel as part of a phase I clinical trial. The treatment, however, was discontinued due to the development of peripheral neuropathy and deep venous thrombosis.

**Figure 1:**
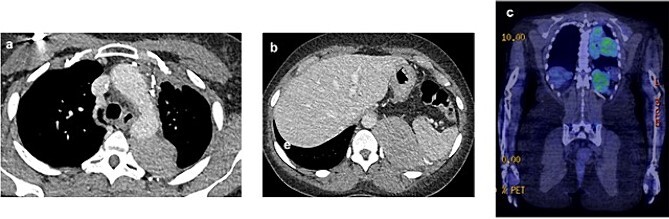
2021 PET and CT scan showing recurrence extension, involving the left parietal pleura (a, c) and the retroperitoneal tissue with spleen capsule (b).

In July 2021, a 18F-fluorodeoxyglucose positron emission tomography integrated with computed tomography revealed active disease in the left hemithorax, with bulky costophrenic and retrocrural disease invading the left aspect of the body and ipsilateral pedicle of the 12th thoracic vertebra, with a maximum standardized uptake value (SUVmax) of 5.9 (Fig. 1).

The case was discussed by a multidisciplinary team, who proposed surgical treatment considering the lack of effective chemotherapy options. Preoperative investigations showed forced expiratory volume in 1 s of 71% of predicted, diffusing capacity for carbon monoxide of 62% of predicted, and maximal oxygen consumption of 78% of predicted. Ventilation/perfusion scintigraphy revealed that the left lung contributed only 4% to the global ventilation, and transthoracic echocardiography demonstrated a left ventricular ejection fraction of 55%, as well as a normal right ventricular function and pulmonary arterial pressures.

**Figure 2:**
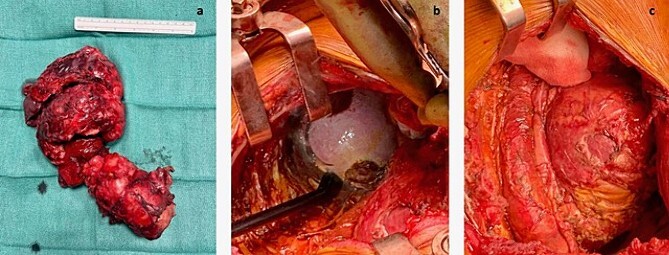
(a) Entire mass. (b) The tumor was attached to the splenic capsule, which was excised. Hemostasis was performed with Aquamantis (Medtronic®). (c) Pericardium after resection of tumor deposit.

In September 2021, she underwent reoperation via a posterolateral thoracotomy for left extra-pleural pneumonectomy, en-bloc resection of the tumor with its retroperitoneal component and the diaphragm, while preserving the left kidney and the spleen, and excision of the metastatic deposit on the 12th thoracic vertebra. The hemidiaphragm was replaced by a 4-mm mesh made of acellular collagen matrix (SurgiMend, Integra LifeSciences, Princeton, NJ, USA) and fixed to the chest wall with permanent multifilament polyester sutures (Ethibond, Ethicon, Bridgewater, NJ, USA). An exploratory laparotomy was subsequently performed to ensure complete resection of the retroperitoneal disease (Fig. 2).

Postoperatively, chest and abdominal drains were removed on the first and fifth days, respectively. She developed mild hospital-acquired pneumonia that was treated with antibiotics and was discharged on the 13th postoperative day.

Histopathological analysis of the resected specimens confirmed recurrent metastatic B2 thymoma, involving lung parenchyma, chest wall, periesophageal fat tissue, pericardium, parietal pleura, and diaphragm (Masaoka–Koga stage IVa), which was completely excised (R0). The case was rediscussed by a multidisciplinary team, with consensus for postoperative radiotherapy. After a 12-months follow-up, that has included physical examination and thoracic imaging investigations, the patient hasn’t showed any sign of disease recurrence.

Written informed consent was obtained from the patient for publication of this case report and any accompanying images.

## Discussion

There are no randomized clinical trials to provide definitive guidance for the management of patients with recurrent thymoma. Depending on the extent of the disease and the patient’s comorbidities and functional status, therapeutic options may include surgery, radiotherapy, and systemic treatments. In the present case, chemotherapy proved ineffective and was associated with significant toxicity. Furthermore, radical radiotherapy was not considered suitable due to the extent of the disease and previous treatment with high radiation dose. Consequently, the patient underwent surgery despite the technical challenges and potential postoperative morbidity associated with the extent and invasiveness of the disease.

Repeat surgery for recurrent thymoma appears to have a survival advantage when compared to other treatment modalities. In a meta-analysis of 8 studies including 205 patients with recurrent thymoma, the average rates of 5- and 10-year overall survival were 70.9% and 49.6%, respectively, for those undergoing surgery (n = 117), as compared to 29.6% and 18.4% for patients treated non-surgically (n = 88) [1]. It is worth noting that completeness of resection, which was reported in five of these studies, was relatively low at 66% of cases. Although these differences in survival may be explained by a greater disease burden in the nonsurgical group, the prolonged survival rates in those treated surgically suggest a potential role for tumor debulking in the management of recurrent thymoma. In a more recent, multicentre study of 155 patients treated for thymoma recurrence, 135 patients (87%) underwent surgery, with complete resection achieved in 109 cases (80.7%) [3]. Surgery demonstrated a survival benefit, with 5- and 10-year overall survival rates of 70.5% and 49.1%, respectively, as compared to 67.3% and 15.1%, respectively, achieved with chemotherapy and/or radiotherapy (*P* = .06).

Completeness of surgical resection after thymoma recurrence is an important prognostic factor. Indeed, in a study of 81 patients who underwent surgery for recurrent thymoma, the 5- and 10-year overall survival rates were 55.9% and 46.5%, respectively, for incomplete resection, as compared to 82.4% and 65.4% for complete resection [4]. Similarly, in the study by Chiapetta *et al*. [3], complete resection of recurrent thymoma was an independent prognostic factor of improved survival (hazard ratio of 1.45, 95% confidence interval of 2.07–10.01; *P* = .01). In our case, a radical surgical approach was adopted, with excision of metastatic deposits and laparotomy to ensure completeness of resection. However, as mentioned above, also tumor debulking may improve locoregional control of the disease and patient survival in highly selected cases. Therefore, surgery might be considered as part of multimodality treatment in cases where complete resection is not technically feasible. In all cases, thorough preoperative evaluation to ensure adequate physiological reserve and optimize medical conditions prior to surgery is paramount for successful surgical outcomes. In cases of extensive and invasive tumors, such as in this case, careful preoperative planning with consideration of previous treatments, including surgery and radiotherapy, is equally important to minimize perioperative complications and increase the likelihood of complete resection.

In conclusion, radical surgery for bulky, invasive recurrent thymoma is safe and can achieve macroscopic complete resection, even in tumors extending through the diaphragm. Careful patient selection is vital for successful outcomes.

## References

[1] Hamaji M , Ali SO, Burt BM. A meta-analysis of surgical versus nonsurgical management of recurrent thymoma. Ann Thorac Surg 2014;98:748–55. 10.1016/j.athoracsur.2014.04.028 24980604

[2] Okumura M , Shiono H, Inoue M, et al. Outcome of surgical treatment for recurrent thymic epithelial tumors with reference to world health organization histologic classification system. J Surg Oncol 2007;95:40–4. 10.1002/jso.20671 17192865

[3] Chiappetta M , Zanfrini E, Giraldi L, et al. Prognostic factors after treatment for iterative thymoma recurrences: a multicentric experience. Lung Cancer 2019;138:27–34. 10.1016/j.lungcan.2019.09.024 31606522

[4] Sandri A , Cusumano G, Lococo F, et al. Long-term results after treatment for recurrent thymoma: a multicenter analysis. J Thorac Oncol 2014;9:1796–804. 10.1097/JTO.0000000000000370 25393792

